# Genomic clustering analysis identifies molecular subtypes of thymic epithelial tumors independent of World Health Organization histologic type

**DOI:** 10.18632/oncotarget.27978

**Published:** 2021-06-08

**Authors:** Sukhmani K. Padda, Yesim Gökmen-Polar, Jessica A. Hellyer, Sunil S. Badve, Neeraj K. Singh, Sumanth M. Vasista, Kabya Basu, Ansu Kumar, Heather A. Wakelee

**Affiliations:** ^1^Stanford University School of Medicine/Stanford Cancer Institute, Stanford, CA, USA; ^2^Indiana University School of Medicine, Indianapolis, IN, USA; ^3^Cellworks Group, San Jose, CA, USA

**Keywords:** thymic epithelial tumor, thymoma, genomics, clustering, computational analysis

## Abstract

Further characterization of thymic epithelial tumors (TETs) is needed. Genomic information from 102 evaluable TETs from The Cancer Genome Atlas (TCGA) dataset and from the IU-TAB-1 cell line (type AB thymoma) underwent clustering analysis to identify molecular subtypes of TETs. Six novel molecular subtypes (TH1-TH6) of TETs from the TCGA were identified, and there was no association with WHO histologic subtype. The IU-TAB-1 cell line clustered into the TH4 molecular subtype and *in vitro* testing of candidate therapeutics was performed. The IU-TAB-1 cell line was noted to be resistant to everolimus (mTORC1 inhibitor) and sensitive to nelfinavir (AKT1 inhibitor) across the endpoints measured. Sensitivity to nelfinavir was due to the IU-TAB-1 cell line’s gain-of function (GOF) mutation in *PIK3CA* and amplification of genes observed from array comparative genomic hybridization (aCGH), including *AURKA*, *ERBB2*, *KIT*, *PDGFRA* and *PDGFB*, that are known upregulate AKT, while resistance to everolimus was primarily driven by upregulation of downstream signaling of *KIT*, *PDGFRA* and *PDGFB* in the presence of mTORC1 inhibition. We present a novel molecular classification of TETs independent of WHO histologic subtype, which may be used for preclinical validation studies of potential candidate therapeutics of interest for this rare disease.

## INTRODUCTION

Thymic epithelial tumors (TETs) are rare tumors that represent a wide spectrum of disease from the indolent thymoma to the more aggressive thymic carcinoma. The World Health Organization (WHO) has categorized these tumors on the basis of immunophenotypic and histopathologic characteristics. Thymoma subtypes include A (including atypical A variant), AB, B1, B2, B3, and other rare categories; in addition, there are TET subtypes of thymic carcinoma and thymic neuroendocrine tumor [1]. However, there are known pitfalls of the classification system that were addressed to some degree in the 2015 4th edition WHO update. These include only modest inter-observer reproducibility [2], intra-tumoral heterogeneity [3], and a weak correlation with prognosis [4]. Therefore, a more robust classification system of TETs is needed.

Identifying the molecular characteristics of TETs has the potential to result in a more refined classification system and subsequent personalization of therapy. As an example, a 9-gene expression signature using real-time quantitative reverse transcription PCR (qRT-PCR) predicted metastatic behavior for thymomas and was a superior prognostic factor to WHO histologic classification [5]. In another study of 34 thymomas, gene expression analysis identified cancer pathways associated with metastases, including those related to amino acid metabolism, cell cycle checkpoint proteins, and Notch signaling [6]. There have been several molecular analyses performed on TETs [7–9], with the The Cancer Genome Atlas (TCGA) reporting on a comprehensive multi-omic analysis of 117 TETs [10]. The most frequently mutated genes included *GTF2I* (39%; majority type A and AB thymomas), *HRAS* (codons 12, 13, 117), *NRAS* (codon 61) and *TP53* (pathogenic loss-of- function). Integrating multi-omic platform results using two different approaches, four subtypes of TETs defined by genomic hallmarks were identified. However, these molecular subtypes correlated to some degree with WHO histologic subtypes as assessed by blinded pathologic review.

Despite the advancement in molecular diagnostics, the molecular aberrations and molecular subtypes discovered from the TCGA have not yet affected therapeutic decisions. Current systemic treatments for TETs rely heavily on chemotherapy options, as studied in small phase II clinical trials and prospective/retrospective cohort studies [11]. Targeted therapies such as sunitinib [12], octreotide [13] and everolimus [14, 15] have resulted in only modest activity for TETs. These results may reflect the unselected patient population enrolled in these studies, including no selection for WHO histologic subtype or molecular aberrations. Despite an increased understanding of TETs, the complex pathology of this rare disease needs to be further elucidated and more biology-driven therapeutic strategies need to be developed.

In this study, we applied computational analyses [16] to the genomic dataset of 102 TETs from the TCGA and the IU-TAB-1 type AB thymoma cell line [17]. The goals were (i) to identify novel molecular subtypes of TETs and examine their association with WHO histologic subtypes, and (ii) to present a proof-of-concept approach of preclinical validation of candidate therapeutics in a molecularly classified cell line for potential further clinical investigation in this rare disease.

## RESULTS

### Genomic clustering analysis identifies TET molecular subtypes independent of WHO histotypes

**Figure 1:**
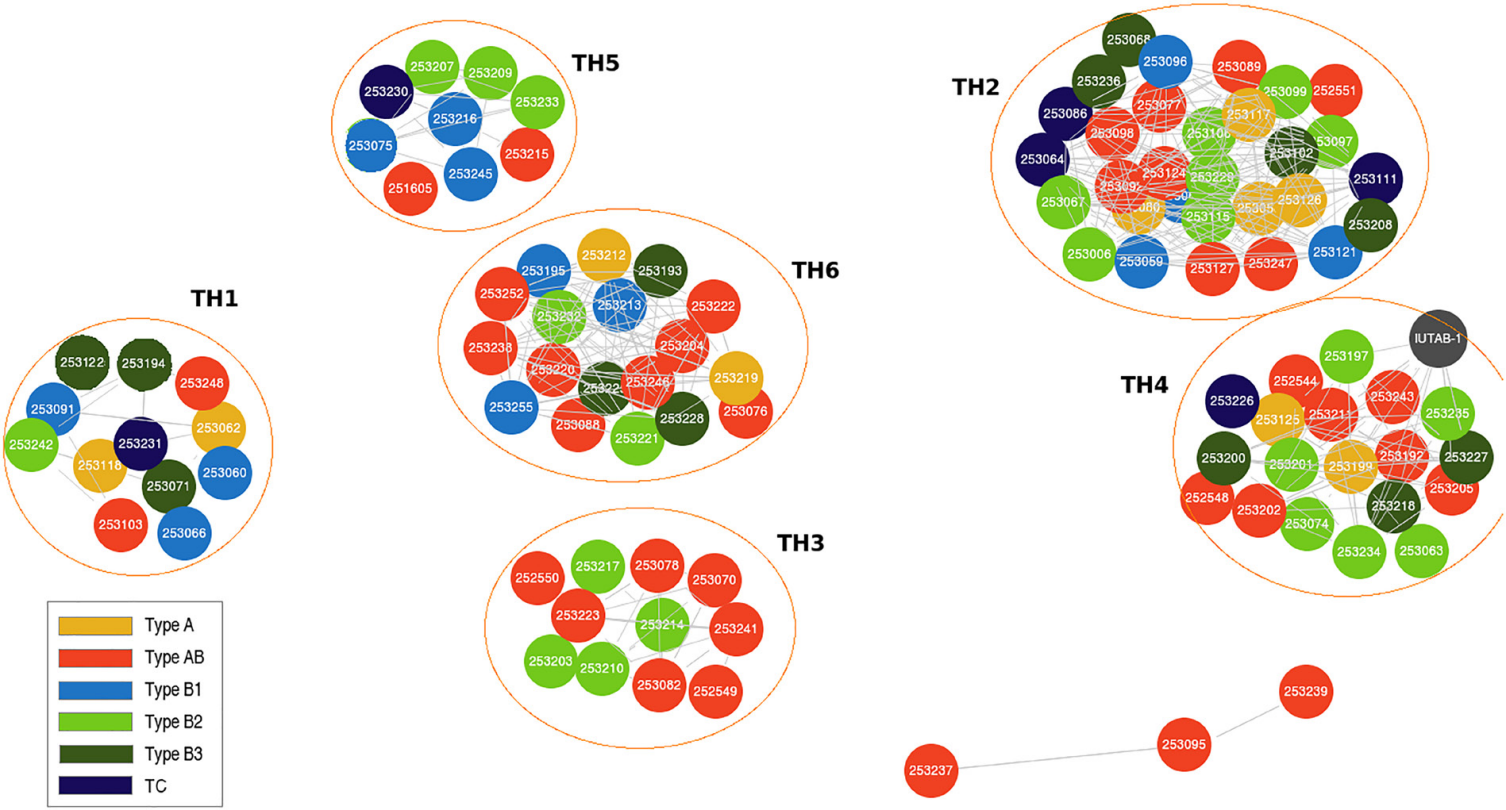
Genomic clustering approach identifies thymic epithelial tumor (TET) molecular subtypes that are independent of World Health Organization (WHO) histotypes.

The 102 evaluable WHO TET histotypes represented in this study from the TCGA database include thymoma type A (10), AB (37), B1 (13), B2 (23), B3 (13) and thymic carcinoma (6). There were no significant differences observed in the demographic, tumor, and treatment characteristics of this sub-cohort when compared to the overall 117 patient TCGA cohort (Supplementary Table 1). Using this clustering analysis [16], 6 unique TET subtypes were identified from the TCGA dataset: TH1 (12), TH2 (30), TH3 (11), TH4 (19), TH5 (9), TH6 (18) and 3 were un-clustered. There was no significant association between the identified TH molecular subtypes and WHO histotypes (Figure 1 and Supplementary Table 2).

### Features of TET molecular subtypes

**Figure 2:**
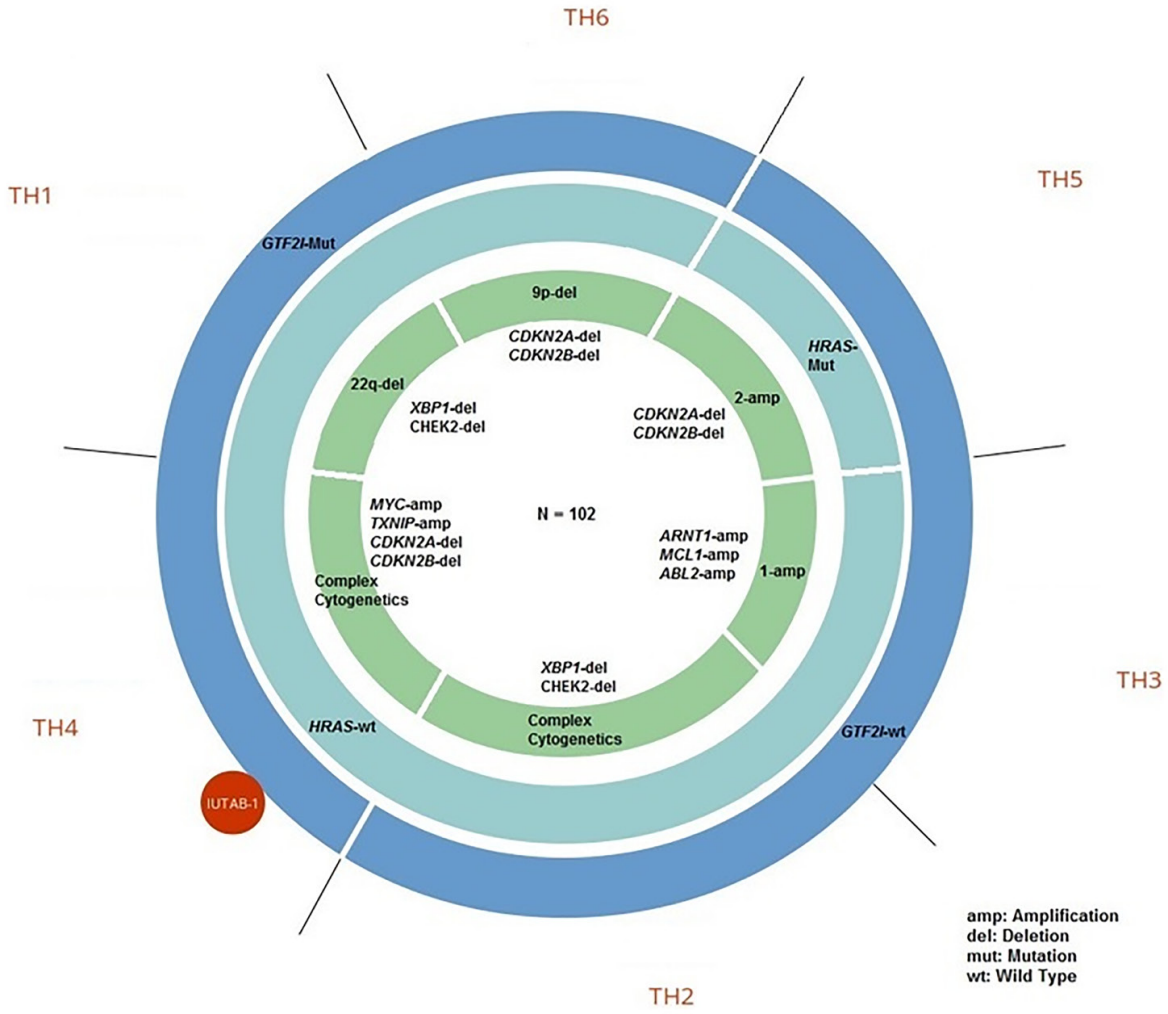
Genomic characteristics of thymic epithelial tumor (TET) molecular subtypes.

Each identified TET molecular subtype was associated with characteristic molecular alterations (Figure 2). The predominant characteristics of each TH subtype includes molecular aberrations present in ≥ 50% of tumors in the subtype (Figure 2 and Table 1). TH subtypes characterized by a *GTF2I* mutation included TH1, TH4, and TH6 while subtypes characterized by *GTF2I* wild type included TH2, TH3, and TH5. Previous analyses have demonstrated recurrent missense *GTF2I* mutations in WHO histologic type A and AB thymomas [7]. In this analysis, subtypes characterized by a *GTF2I* mutation had the following molecular features that predominated: chromosome 22q deletion (del) in TH1 (e.g., *XBP1, CHEK2, NF2, MAPK1*); complex cytogenetics in TH4 (e.g., *MYC* amplification, *TXNIP1* amplification and *CDKN2A/B* del); and chromosome 9p del in TH6 (e.g., *CDKN2A/B, VCP, TLN1, PAX5*). Among TH subtypes characterized by *GTF2I* wild type, the following molecular features predominated: complex cytogenetics in TH2 (i.e., multiple cytogenetic aberrations not identified in a specific pattern); chromosome 1 amplification in TH3 (e.g., *MCL1, ARNT, ABL2, PTPRC, GADD45A*); and *HRAS* mutation, chromosome 2 amplification (e.g., *ERBB4, IRS1*) and *CDKN2A/B* del in TH5.

**Table 1:** Molecular aberrations in thymic epithelial tumor (TET) molecular subtypes

Subtype	*GTF2I* mutation	*HRAS* mutation	Chr9p-deletion^a^	Chr22q-deletion^b^	Complex-Cytogenetics^c^	Chr1-amplification^d^	Chr2-amplification^e^
TH1 (*n* = 12)	**58.3%** (7)	0%	16.7% (2)	**50%** (6)	16.7% (2)	8.3% (1)	8.3% (1)
TH2 (*n* = 30)	13.3% (4)	0%	13.3% (4)	10% (3)	**70%** (21)	0%	3.3% (1)
TH3 (*n* = 11)	18.2% (2)	0%	9.1% (1)	9.1% (1)	9.1% (1)	**54.5%** (6)	0%
TH4 (*n* = 19)	**73.7%** (14)	0%	15.8% (3)	15.8% (3)	**63.2%** (12)	0%	0%
TH5 (*n* = 9)	0%	**55.6%** (5)	0%	0%	11.1% (1)	11.1% (1)	**55.6%** (5)
TH6 (*n* = 18)	**66.7%** (12)	0%	**55.6%** (10)	5.6% (1)	11.1% (2)	5.6% (1)	16.7% (3)

Percentages are row percentages. Bolded numbers indicate a prevalence of ≥ 50% of the molecular aberration in the subtype. *Chr*: *chromosome.* ^a^Chromosome 9p includes *CDKN2A/B*, *VCP*, *TLN1*, *PAX5*. ^b^Chromosome 22q includes *XBP1*, *CHEK2*, *NF2*, *MAPK1*. ^c^Complex cytogenetics includes the following genes of relevance for TH4 subtype: *MYC* amplification, *TXNIP1* amplification, *CDKN2A/B* deletion. There was no specific pattern observed for the TH2 subtype. ^d^Chromosome 1 includes *MCL1*, *ARNT*, *ABL2, PTPRC, GADD45A*. ^e^Chromosome 2 includes *ERBB4*, *IRS1*.

### IU-TAB-1 cell line characteristics and candidate therapeutic *in vitro* experiments

The IU-TAB-1 cell line consists of a gain-of-function mutation in *PIK3CA* and an amplification of genes including *AURKA, ERBB2, KIT, PDGFRA* and *PDGFB*, all of which are known to activate the AKT pathway [18–24]. Presence of *FHIT* and *CDH1* deletion in the IU-TAB-1 cell line may also upregulate the beta catenin pathway [25, 26].

**Figure 3:**
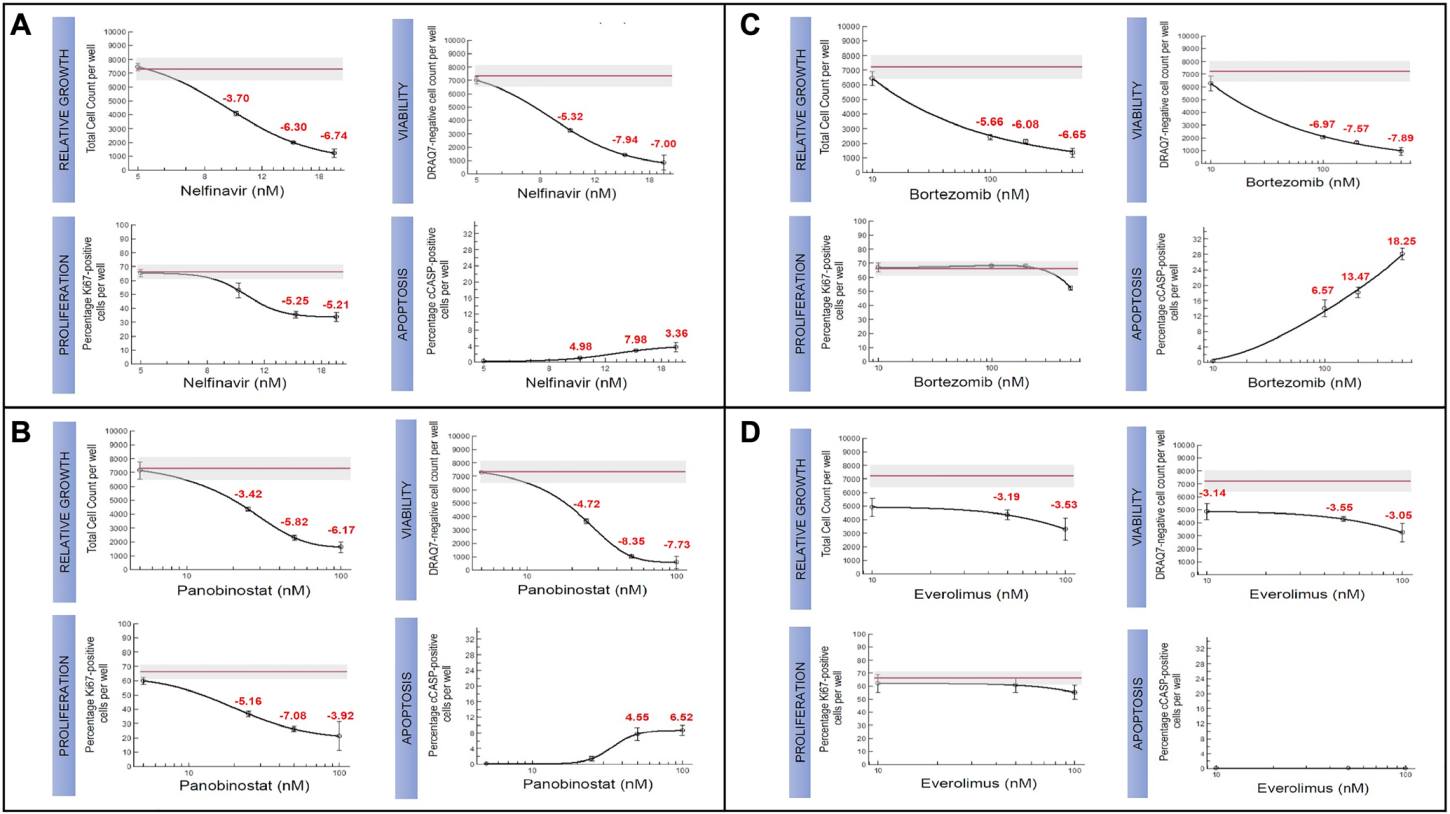
Candidate therapeutics tested for TH4 subtype in IU-TAB-1 cell line. Experimental dose-response curves assessing relative growth (Hoechst staining of DNA), proliferation (Ki67), viability (DRAQ7 assay), and apoptosis (caspase) for (**A**) Nelfinavir, (**B**) Panobinostat, (**C**) Bortezomib, and (**D**) Everolimus. The red line in the experimental graphs indicate DMSO treated control.

The IU-TAB-1 cell line clustered into the TH4 molecular subtype (Figure 2). Therefore, the IU-TAB-1 cell line was used for *in vitro* candidate therapeutic testing of the TH4 subtype, including with nelfinavir (AKT1 inhibitor) [27], panobinostat (histone deacetylase [HDAC] inhibitor) [28], bortezomib (proteasome inhibitor) [29] and everolimus (mTORC1 inhibitor) [14] (Figure 3A–3D).

Nelfinavir [27], had significant impact on outcomes of relative growth (IC50 ~10 uM), proliferation, viability, and apoptosis in the IU-TAB-1 cell line (Figure 3A). The sensitivity of the AKT1 inhibitor is due to the presence of a gain-of function (GOF) mutation in *PIK3CA* and amplification of genes observed from array comparative genomic hybridization (aCGH) that result in upregulation of AKT, including *AURKA, ERBB2, KIT, PDGFRA* and *PDGFB* [18–24].

Panobinostat [28] inhibited relative growth (IC50 ~30 nM), proliferation and viability and increased apoptosis in the IU-TAB-1 cell line (Figure 3B). The sensitivity of the HDAC inhibitor is possibly due to deletion of *ARID1A*, an epigenetic regulator encoding a subunit of SWI/SNF chromatid-remodeling complex, which has been linked to the upregulation of *HDAC6* [30, 31]. It was also observed that HDAC inhibition significantly suppressed the tumor growth in an *ARID1A*^−/−/^ *PIK3CA*^MUT^ genetic clear cell ovarian tumor mouse model and this similar molecular condition of *ARID1A* deletion and *PIK3CA* mutation is present in the IU-TAB-1 cell line [30].

Bortezomib [29] had variable impact on outcomes measured in the IU-TAB-1 cell line, with resistance noted in proliferation endpoints but sensitivity noted in relative growth, viability and apoptosis endpoints (Figure 3C). The reason for IU-TAB-1 resistance to the anti-proliferative effect of bortezomib was due to deletion of *CDH1* and *FHIT*, which are known to upregulate the beta catenin pathway, a key protein in the canonical Wingless/int (Wnt) pathway [25, 26], and subsequently increase cell proliferation and G2 to M cell cycle transition via *CCND1* [32, 33]. Bortezomib was also observed to lead to accumulation of beta catenin protein in a dose- and time-dependent manner without changing the mRNA level in multiple myeloma cell lines, suggesting the effect was at the post-transcriptional level and this accumulation was associated with bortezomib resistance [34]. In addition, there is loss of *SPEN* in the IU-TAB-1 cell line, a transcriptional co-repressor that directly binds and negatively regulates *RBPJ* [35]. This can result in upregulation of the NOTCH pathway via formation of the Notch transcription activation complex (NTC), which has been demonstrated to contribute to bortezomib resistance in multiple myeloma [36].

It was interesting to note that everolimus, mTORC1 inhibitor, with clinical activity previously noted in a subset of patients with TETs [14, 15], had no effect on relative growth, proliferation, viability, and apoptosis in the IU-TAB-1 cell line. There was no evidence of apoptosis even as concentrations approached 100 nM (Figure 3D). Despite the *PIK3CA* mutation and other aberrations such as *AURKA* amplification resulting in downstream upregulation of the *AKT1* pathway [19], the resistance to everolimus was due to amplification of *KIT*, *PDGFRA*, and *PDGFB* observed in the IU-TAB-1 cell line. mTOR inhibition can result in upregulation of pro-survival signaling downstream of *KIT*, *PDGFRA* and *PDGFB* via release of mTOR-mediated negative feedback loops (e.g., via GRB10) [37–39], possibly accounting for resistance to everolimus.

## DISCUSSION

In this study, we present an updated molecular classification system for TETs using a correlation clustering method of molecular data, including genomic aberrations and copy number variations [16]. There are significant limitations to the current WHO classification system for TETs, which is predicated upon epithelial cell morphology and lymphocyte abundance [1–4]. The current classification system also does not provide insight into the molecular characteristics of TETs and thus is limited in its ability to inform potential therapeutic decisions.

In our study, we identified six molecular subtypes of TETs (TH1-TH6), and notably, they were independent of WHO histotypes, which is in contrast to prior TCGA reports [10, 40]. In the cluster-of-clusters-assignments (COCA) analysis from the primary TCGA publication by Radovich et al, data from somatic copy number variation (sCNV), mRNA, miRNA, DNA methylation, and reverse phase protein array (RPPA) data was used to identify four molecular subtypes. These molecular subtypes strongly correlated with WHO histotypes including type B, thymic carcinoma, type AB, and a mix of types A and B. In a complementary approach to COCA known as TumorMap, incorporating each single platform analysis performed along with multi-platform PARADIGM analyses (copy number plus gene expression data), four molecular subtypes were identified. These also strongly correlated with WHO histotypes, including A-like, AB-like, B-like, and C-like clusters. In a separate publication, Lee et al used a decision tree approach and data from DNA mutational analyses, unsupervised clustering of mRNA expression data, and sCNV, to propose four molecular subtypes from the TCGA cohort that also correlated with WHO histotypes [40]. In our clustering analysis, three of the 102 tumors were un-clustered due to insufficient number of genomic alterations/CNVs. In addition, 15 tumors from the original TCGA dataset were not evaluable upfront due to this reason, representing a limitation of this analysis. Despite the TCGA being a relatively large dataset with comprehensive multi-omic analyses for this rare disease, the representation of certain histotypes was limited (e.g., type A, B1, B3, and thymic carcinoma) and the stage of tumors was biased towards early stage (e.g., I, IIA, IIB).

Targeted therapies have been and are being actively examined in TETs, with almost all studies not including biomarker selection *a priori*. For example, in a phase 2 study of everolimus in TETs, the overall disease control rate (DCR) was 88% (thymoma 93.8%; thymic carcinoma 77.8%) and the response rate was 12% (thymoma 9.4%; thymic carcinoma 16.7%) in 44 evaluable patients [14]. However, this clinical trial did not perform molecular profiling of the tumors. A retrospective analysis of a small cohort of patients (*N* = 15) from our institution with advanced thymic tumors treated with everolimus also failed to identify molecular biomarkers of response. There were several patients with durable responses to everolimus who had tumor mutations in the *fibroblast growth factor family of receptors* (*FGFR*). However, the small sample size made it difficult to draw definitive conclusions about the predictive nature of these alterations [15]. There is likely limited utility of a single genomic aberration as a predictive biomarker for treatment in patients with TETs, highlighting the importance of alternative methods for treatment selection. For example, in a phase 2 study of sunitinib, a multi-targeted tyrosine kinase inhibitor of VEGFR, KIT, and PDGFR, there was notable activity in patients with thymic carcinoma, with a DCR of 91% and response rate of 26% in 23 evaluable patients [12]. Molecular profiling of tumors was performed in 22 patients (13 with thymic carcinoma) and there was no association with any specific mutation and response, although no *KIT* mutations were identified. In a real world study of sunitinib in TETs, *KIT* genotyping was performed in 8 of 28 cases and 3 had a *KIT* mutation without a clear association with response [41].

The hope is that a novel classification of TETs using genomic information may render more precise therapy selection for patients in the future, as there are known challenges of developing new therapies in a rare disease. In our study, we were able to test candidate therapeutics of interest for the novel TH4 subtype since the IU-TAB-1 cell line clustered into the TH4 subtype. *In vitro*, there was sensitivity noted for nelfinavir (AKT1 inhibitor) [27], panobinostat (HDAC inhibitor) [28], and bortezomib (proteasome inhibitor) [29], while resistance was noted for everolimus (mTORC1 inhibitor). There were genomic explanations for the therapeutic sensitivities observed in this molecular cluster. These findings may be relevant, as some of the candidate therapeutics tested *in vitro* in our study are similar to completed and ongoing targeted therapy studies in patients with TETs (e.g., HDAC inhibitor belinostat [42], PI3K inhibitor buparlisib [43]). In addition, given the resistance to everolimus, patients with tumors that cluster into the TH4 subtype, may not benefit from everolimus.

Unfortunately, preclinical models for TETs are limited to a handful of cell lines [17, 43] and clinical trials are limited to single-arm phase II studies. This computational analysis involves data available from tests used routinely in the clinical setting such as targeted next generation sequencing assays, including gene mutations, copy number variations, and chromosomal aberrations. In our study, computational analysis of the TCGA dataset reveals an updated molecular classification of TETs and identifies 6 unique molecular subtypes; importantly, these subtypes are independent of WHO histologic subtypes. Only the IU-TAB-1 cell line underwent clustering analysis and was used for preclinical testing of candidate therapeutics for one of the six identified molecular subtypes from the TCGA. Although the IU-TAB-1 cell line reflects the predominant histotype of type AB thymoma from the TCGA dataset and has been extensively characterized by both whole exome sequencing (WES) and aCGH [17], its inherent limitations include generation from an early stage tumor and representation of a histotype that portends a better prognosis and less metastatic potential [1–4]. Therefore, future work should involve further genomic characterization and clustering analyses of TETs, particularly from metastatic tumors, and generation of diverse TET cell lines to evaluate whether this proof-of-concept approach of preclinical candidate therapeutic testing in molecularly classified cell lines is promising for clinical translation in patients with this rare disease.

## MATERIALS AND METHODS

### Computational analysis of TCGA dataset and IU-TAB-1 cell line

Computational analysis [16] was applied to data from WES of 102 evaluable patients with TETs from the publically available TCGA (includes total of 117 patients) [10] and also to data from WES and aCGH of the IU-TAB-1 (type AB thymoma) cell line [17]. Specifically, this included CNVs and genomic mutations/aberrations. A subset of 15 patients with TETs were not evaluable due to an insufficient quantity of genomic aberrations identified below the threshold of the computational analysis. It is possible that data from more comprehensive whole genome sequencing would have made these tumors evaluable.

Each tumor in the TCGA was classified based on the presence or absence of each identified mutation/aberration and CNV (i.e., genomic mutation handle). Overlapping mutations and CNVs were iteratively classified as a molecular subtype based on the cumulative frequency ranked method (Supplemental Methods) [16]. Clusters of overlapping high-frequency mutations/aberrations and CNVs were identified, with clusters of greater than 5 tumors considered to be a significant molecular subtype.

### Cell culture and *in vitro* assays

The IU-TAB-1 cell line was established from a patient with stage II type AB thymoma and characterized as described previously [17]. The cell line had the same passage as in the aforementioned publication. In this study, the IU-TAB-1 cell line was identified to be part of the TH4 cluster. *In vitro* experiments of candidate therapeutics, chosen based on their mechanism of action and ongoing or completed clinical trials in patients with TETs, were performed on the IU-TAB-1 cell line and included measurements of cell viability [44], proliferation (Ki67), apoptosis [45], and growth (i.e., total cell count) [46]. The known clinical pharmacokinetics of each drug, including the clinically observed concentration range and maximum concentration (Cmax) value, informed the experimental design.

## SUPPLEMENTARY MATERIALS



## Abbreviations

ABL2: ABL proto-oncogene 2, non-receptor tyrosine kinase
AKT1: AKT serine/threonine kinase 1
ARID1A: AT-rich interaction domain 1A
ARNT: aryl hydrocarbon receptor nuclear translocator
AURKA: aurora kinase A
CCND1: cyclin D1
CDH1: cadherin 1
CDKN2A: cyclin dependent kinase inhibitor 2A
CDKN2B: cyclin dependent kinase inhibitor 2B
CHEK2: checkpoint kinase 2
ERBB2: erb-b2 receptor tyrosine kinase 2
ERBB4: erb-b2 receptor tyrosine kinase 4
FGFR: fibroblast growth factor receptor
FHIT: fragile histidine triad diadenosine triphosphatase
GADD45A: growth arrest and DNA damage inducible alpha
GRB10: growth factor receptor bound protein 10
GTF2I: general transcription factor III
HDAC: histone deacetylase
HDAC6: histone deacetylase 6
HRAS: HRas proto-oncogene, GTPase
IRS1: insulin receptor substrate 1
KIT: KIT proto-oncogene, receptor tyrosine kinase
MAPK1: mitogen-activated protein kinase 1
MCL1: MCL1 apoptosis regulator, BCL2 family member
mTORC1: mammalian target of rapamycin complex 1
MYC: MYC proto-oncogene, bHLH transcription factor
NF2: neurofibromin 2
NRAS: NRAS proto-oncogene, GTPase
PAX5: paired box 5
PDGFB: platelet derived growth factor subunit B
PDGFR: platelet derived growth factor receptor
PDGFRA: platelet derived growth factor receptor alpha
PIK3CA: phosphatidylinositol-4,5-bisphosphate 3-kinase catalytic subunit alpha
PTPRC: protein tyrosine phosphatase receptor type C
RBPJ: recombination signal binding protein for immunoglobulin kappa J region
SPEN: spen family transcriptional repressor
TLN1: talin 1
TP53: tumor protein p53
TXNIP1: thioredoxin interacting protein
VCP: valosin containing protein
VEGFR: vascular endothelial growth factor receptor
XBP1: X-box binding protein 1
