# Supplementary material for: Genomic clustering analysis identifies molecular subtypes of thymic epithelial tumors independent of World Health Organization histologic type
Source: Oncotarget. 2021 Jun 8;12(12):1178–86. doi: 10.18632/oncotarget.27978 (PMC8202771; doi:10.18632/oncotarget.27978)
Supplement: Supplementary file 1 [file oncotarget-12-1178-s001.pdf]

Genomic clustering analysis identifies molecular subtypes of thymic epithelial tumors independent of World Health Organization histologic type

SUPPLEMENTARY MATERIALS

A two-step process was followed to form the molecular subtype clusters of TETs. In the first step, the data was sourced from The Cancer Genome Atlas (TCGA) database. Genomic data from 102 TETs was identified and was used to generate a normalized feature matrix, including presence or absence of a genomic mutation and copy number variation (CNV) handle. The optimal number for 'k' was iteratively identified using the elbow method [1, 2]. The elbow method is a heuristic method of interpretation and validation of consistency within cluster analysis designed to help find the appropriate number of clusters in a dataset. The algorithmic aim was to choose the smallest value of 'k' within the TCGA dataset that had minimal error deviation function. In the second step, using the optimal 'k' value, the k-means algorithm identified clusters by iteratively improvising the centroid mean selection until there was no delta change in the cluster formation observed between three consecutive iterations. This step was critical in assessing that the identified clusters were distinct from each other. As a result of this clustering methodology, each identified cluster had overlapping high frequency mutations/aberrations and CNVs.

SUPPLEMENTARY REFERENCES

1. Demidenko E. The next-generation K-means algorithm. Stat Anal Data Min. 2018; 11:153–66. https://doi.org/10.1002/sam.11379. [PubMed]
2. Syakur MA, Khotimah BK, Rochman EMS, Satoto BD. Integration K-Means Clustering Method and Elbow Method For Identification of The Best Customer Profile Cluster. IOP Conference Series: Materials Science and Engineering. 2018; 336:012017.

Supplementary Table 1: Characteristics of thymic epithelial tumors from this sub-cohort (n = 102) compared to overall cohort (n = 117) from The Cancer Genome Atlas

Parameter	Sub-cohort Total *n* (%)	TCGA cohort Total *n* (%)	*P*-Value[a]
**Total Number**	**102**	**117**	
**Age-Years**, Median (range)	60.5 (17–84)	60 (17–84)	0.708
**Gender**			
Male	47 (46)	61 (52)	0.231
Female	55 (54)	56 (48)	
**Race**			
White	79 (77)	97 (83)	0.424
Black OR African American	6 (6)	6 (5)	
Asian	15 (15)	12 (10)	
Data Missing	2 (2)	2 (2)	
**Ethnicity**			
Hispanic OR Latino	10 (10)	9 (8)	0.609
Not Hispanic OR Latino	82 (80)	94 (80)	
Data Missing	10 (10)	14 (12)	
**Masaoka Stage**			
I	33 (32)	36 (31)	0.622
IIa	37 (36)	39 (33)	
IIb	15 (15)	19 (16)	
III	14 (14)	15 (13)	
IVa	1 (1)	1 (1)	
IVb	1 (1)	5 (4)	
Data Missing	1 (1)	2 (2)	
**Histologic Subtype**			
Thymoma Total	96	105	
Thymoma A	10 (10)	10 (9)	0.795
Thymoma AB	37 (36)	48 (41)	
Thymoma B1	13 (13)	12 (10)	
Thymoma B2	23 (23)	25 (21)	
Thymoma B3	13 (13)	10 (9)	
Thymic Carcinoma Total	6	10	
Squamous cell carcinoma	3 (3)	4 (3)	0.855
Undifferentiated Carcinoma	1 (1)	4 (3)	
Large cell neuroendocrine carcinoma	1 (1)	1 (1)	
Thymic carcinoma, NOS	1 (1)	1 (1)	
Micronodular thymoma	0	2 (2)	
**Adjuvant Radiation Therapy**			
Yes	33 (32)	39 (33)	0.550
No	69 (68)	78 (67)	
**History of Myasthenia Gravis**			
Yes	31 (30)	32 (27)	0.615
No	70 (69)	84 (72)	
Data Missing	1 (1)	1 (1)	

[a]Paired *t*-test was used to compare continuous variables and Chi-square test was used to compare categorical variables. Column percentages may not add up to 100% due to rounding.

Supplementary Table 2: Distribution of World Health Organization (WHO) histologic subtype per thymic epithelial tumor (TET) identified molecular cluster

	TH1<br>*n* (%)	TH2<br>*n* (%)	TH3<br>*n* (%)	TH4<br>*n* (%)	TH5<br>*n* (%)	TH6<br>*n* (%)
**Type A**	2 (16.7)	4 (13.3)	0 (0)	2 (10.5)	0 (0)	2 (11.1)
**Type AB**	2 (16.7)	8 (26.7)	7 (63.6)	7 (36.8)	2 (22.2)	8 (44.4)
**Type B1**	3 (25)	4 (13.3)	0 (0)	0 (0)	3 (33.3)	3 (16.7)
**Type B2**	1 (8.3)	7 (23.3)	4 (36.4)	6 (31.6)	3 (33.3)	2 (11.1)
**Type B3**	3 (25)	4 (13.3)	0 (0)	3 (15.8)	0 (0)	3 (16.7)
**Thymic Carcinoma**	1 (8.3)	3 (10)	0 (0)	1 (5.3)	1 (11.1)	0 (0)
**Total**	12	30	11	19	9	18

No significant difference observed in distribution of WHO histotypes among the identified molecular subtypes (Chi Square test; $p = 0.284$).
